# The Role of Virtual Communities in Gambling and Gaming Behaviors: A Systematic Review

**DOI:** 10.1007/s10899-020-09946-1

**Published:** 2020-04-18

**Authors:** Anu Sirola, Nina Savela, Iina Savolainen, Markus Kaakinen, Atte Oksanen

**Affiliations:** 1 Faculty of Social Sciences, Tampere University, 33014 Tampere, Finland; 2 Institute of Criminology and Legal Policy, University of Helsinki, Helsinki, Finland

**Keywords:** Gambling, Gaming, Virtual communities, Social media

## Abstract

Gambling opportunities are facilitated by the growth of the Internet and social media platforms. Digital games also increasingly include monetary features, such as microtransactions, blurring the line between gambling and gaming. The Internet provides a variety of virtual communities for gamblers and gamers, but comprehensive research on these communities and their relevance in gambling and monetary gaming behaviors remains scarce. This paper summarizes research of online gambling and monetary gaming communities based on a systematic literature review. A systematic literature search was conducted from five databases: Scopus, Web of Science, PsycINFO, Social Science Premium Collection, and EBSCOhost. The search was limited to empirical articles that focused on gambling or gaming involving money and examined online interaction between gamblers or gamers. Preliminary search resulted in 1056 articles, from which 55 were selected for the analyses based on pre-determined criteria. According to results, online communities serve different functions in gambling and gaming behaviors. Gambling communities are typically forums for discussing and sharing gambling experiences, strategies, and tips as well as gambling problems, while gaming communities are inherently embedded inside a game being an essential part of the gaming experience. Identification with virtual communities influences gambling behavior and monetary gaming behavior through mechanisms of perceived norms, social influence, and community feedback. Whereas some gambling communities may provide protection from excessive gambling habits, gaming communities seem to solely motivate gaming behavior and purchase intentions. The role of online communities should be acknowledged in prevention and treatment of gambling and gaming problems.

## Introduction

The Internet and social media have facilitated and extended gambling opportunities via exponential growth of online gambling platforms. Consequently, social media users are increasingly exposed to gambling content and gambling-like activities in social media (King and Delfabbro 2016). At the same time, gambling problems are growing globally (Calado and Griffiths 2016). Online games and video games increasingly include gambling-like and monetary features, such as microtransactions (Jacques et al. 2016; H. S. Kim et al. 2017; King et al. 2015), blurring the line between gambling and gaming. Gambling and gambling-like behaviors can be detrimental particularly when excessive, and lead to severe and long-lasting problems, such as economic difficulties (Oksanen et al. 2018).

In addition to gambling and gaming platforms, the Internet also offers social environments for gamblers and gamers, such as discussion forums and in-game interaction tools. These kinds of consumption-related online communities (Kozinets 1999) and their social aspects may have an important role in gambling and monetary gaming behaviors, but comprehensive research on these communities and their relevance to users remains scarce. In this systematic literature review, we aim to summarize earlier research on online gambling and gaming communities and their role in gambling and monetary gaming behaviors.

### The Blurring Line Between Gambling and Gaming

Gambling and gaming have been traditionally perceived as distinct activities. King et al. (2015) roughly distinguish gambling and gaming based on their central features: gambling is characterized by its risk-involving, chance-determined outcomes and monetary features, such as wagering and betting mechanisms, whereas gaming is characterized by interactive, skill-based play and contextual relevance in game progress and success. However, these boundaries have become more and more blurred, partly due to technological divergence.

Digital games increasingly utilize monetary features, typically microtransactions, as revenue models. Microtransactions are needed, for example, to get additional features or better equipment in a game. Also, so called “loot boxes” have become common particularly in video games, sharing the chance-determined features of gambling. Loot boxes are virtual entities that contain randomized items (e.g., weapons or other equipment) and can be paid with real-world money. Recent research found that spending on loot boxes was associated with problematic gambling (Zendle and Cairns 2018). It has also been suggested that due to many similarities between gambling and gaming, playing video games would increase a desire to gamble; but recent research has not fully supported this (Forrest et al. 2016; Macey and Hamari 2018).

In addition to video games, online games increasingly include gambling-like features. For example, social media sites, such as Facebook, include social games that simulate gambling activities like poker, roulette, or slot machines (Calado et al. 2018; Jacques et al. 2016; King et al. 2014). Although these types of games are often perceived as harmless and safe alternatives for real-money gambling, their gambling-like characteristics may also trigger motivation for real gambling (King et al. 2014) and teach mechanisms of gambling to children and adolescents (King et al. 2010). Moreover, while “free-to-play” games do not initially require real-money use, they typically encourage players to make in-game purchases (i.e., microtransactions) to get access to additional features (H. S. Kim et al. 2017; Paavilainen et al. 2013). The aforementioned studies demonstrate that gambling and gaming can no longer be perceived as fully distinct activities. Rather, they increasingly share common characteristics related to gambling-like mechanisms.

### Online Communities: Social Dimension of Gambling and Gaming

Humans have a basic need for social belonging and relatedness (Baumeister and Leary 1995; Deci and Ryan 2000), which is one of the reasons behind the success of online communities and social media (Keipi and Oksanen 2014; McKenna and Bargh 1999; Reich and Vorderer 2013; William et al. 2000). Following Kozinets’ (1999) fundamental definition, virtual communities (i.e., online communities) consist of groups of people sharing social interactions, social ties, and virtual spaces for interactions. Communities are characterized by shared interests, goals, and norms that unite like-minded individuals (Preece 2000; Rheingold 1993). Indeed, in a virtual environment people have a tendency toward homophily, that is, to seek for and interact with similar others (Centola and van de Rijt 2015; McPherson et al. 2001).

Identifying with a virtual community consisting of like-minded people may have important consequences for a user (Kaakinen et al. 2020). Identifying with the community’s shared social identity and internalizing its group norms affect user behavior (Zhou 2011). Moreover, social media research shows that people often rely on information and content provided by their in-group members (Flanagin et al. 2014). Particularly when talking about potentially addictive behaviors, identifying with an online community can influence intentions and attitudes toward harmful direction and normalize maladaptive behavior (Oksanen et al. 2016). However, online communities and shared identity may also be beneficial in overcoming an addiction (McNamara and Parsons 2016).

In terms of gambling and gaming, online communities cover various kinds of virtual spaces, such as discussion forums and social network sites, where gamblers and gamers can interact with other gamblers and gamers. However, social interaction is not limited to distinct online platforms, as games often also include in-game interactive tools. Video games, in particular, are typically formed around interactive elements, such as communicating with one’s team members during the game, which are not essentially the case in traditional forms of gambling activities (Cole and Griffiths 2007; King et al. 2015). In particular, Massively Multiplayer Online Role-Playing Games (MMORPGs) are characterized by their community aspects and joint playing. In MMORPGs, gaming typically takes place in “guilds” that can be defined as long-lasting social structures where players are interdependent on each other’s contribution (Zhong 2011). Guild playing is also important in terms of a player’s game-related social identity (Guegan et al. 2015). In this review, we examine these different virtual spaces and their role in gambling and monetary gaming behaviors in more depth.

### Current Study

The aim of this study is to bring additional insight into the gambling and gaming phenomena by investigating the role of online communities in gambling and monetary gaming behaviors. In this review, we adopt a loose definition of online communities (see Kozinets 1999; Preece 2000; Rheingold 1993) to cover various kinds of interactive online platforms for gamblers and gamers.

Some systematic reviews close to our topic have been conducted, for example in terms of online game communities (Warmelink and Siitonen 2013) and user participation in different online communities (Malinen 2015). However, our focus lies in the social aspects of the online gambling and monetary gaming phenomena. Thus, we aim to synthesize empirical evidence of the key characteristics and the roles of virtual gambling and gaming communities in gambling and monetary gaming behaviors. Since we are specifically interested in the role of virtual communities in gambling and gambling-like behaviors, we narrow our perspective of gaming to cover only gaming involving money. We believe this is reasonable when examining gaming alongside gambling. As we argued earlier, it is meaningful to include both gambling and gaming phenomena because of their combined monetary features; but, as such, we are also able to compare possible differences among these communities. Consequently, the more general role of online communities in gaming is out of our focus.

Our research questions are as follows:

- *RQ1* What is the role of virtual gambling communities in gambling behavior?
- *RQ2* What is the role of virtual gaming communities in monetary gaming behavior?
- *RQ3* Are there notable qualitative differences between virtual gambling and gaming communities?

## Method

### Data Collection

To answer our research questions, we conducted a conceptual review with a systematic data collection process (e.g., Petticrew and Roberts 2006, p. 39). The data were collected in two phases: The original search was conducted in July 2018 from five comprehensive databases: Scopus (Elsevier), Web of Science (Clarivate), PsycINFO (APA), Social Science Premium Collection (ProQuest), and EBSCOhost (EBSCO) with all databases selected. The search engines were set to search hits from abstracts, titles, and keywords using the same search phrase in each database: (gambl* OR gaming OR gamer) AND (internet OR online OR virtual OR digital) AND (“online communit*” OR “virtual communit*” OR “online group*” OR “virtual group*” OR “online discuss*” OR “chat room*” OR “online social network*” OR “forum*”). In addition to author keywords, the database keyword indexes were included in the search fields when applicable. Due to the vast amount of magazines and other irrelevant sources in Social Science Premium Collection and EBSCOhost, only scholarly or academic journals were selected using the filtering options within the search engines. We used no other limits in the search engines, for example, year or language. After removing duplicates, the database search resulted in 885 articles.

In order to keep the data up-to-date, we conducted an additional literature search in February 2020, following the same steps and guidelines established in 2018. The search was conducted from the same five databases: Scopus (Elsevier), Web of Science (Clarivate), PsycINFO (APA), Social Science Premium Collection (ProQuest), and EBSCOhost (EBSCO). In databases, the publication time was limited to cover years 2018-2020. After removing duplicates and overlaps with data gathered in 2018, the additional database search resulted in 171 articles.

In both data collection phases, studies were included based on the following criteria. (1) The article empirically examines participation or social interaction in online communities or networks related to gambling or gaming involving money. Participation or interaction can include aspects such as participation frequency, motivation, level of identification, or shared content between users. (2) The article empirically examines behavioral factors associated with participation or social interaction in online community or networks related to gambling or gaming involving money. Behavioral factors can include aspects such as virtual purchase behavior, frequency of gambling or gaming behaviors or other kinds of gambling and gaming behaviors involving money. Consequently, studies were excluded if they did not mention gambling, monetary gaming, or social interaction between gamblers and gamers; if they were theoretical articles or literature reviews; book or conference introductions; or were not published in English.

**Fig. 1:**
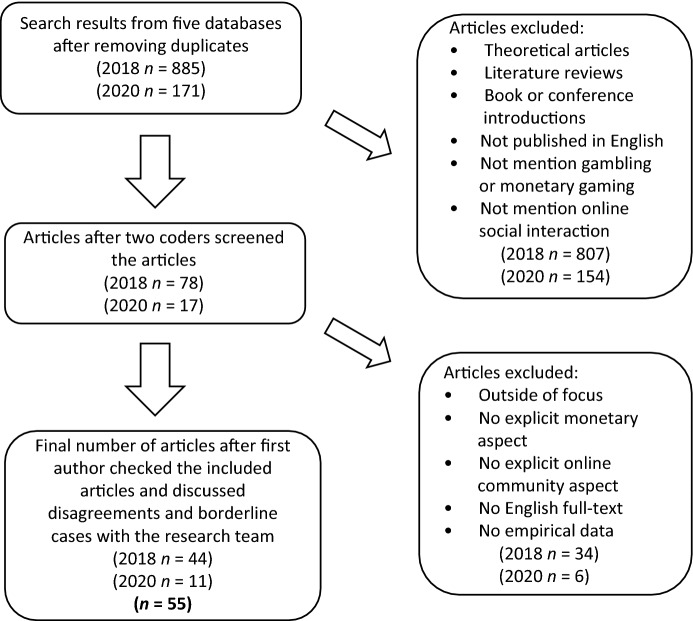
Data collection and selection process accomplished in two phases in 2018 and 2020

In the first data collection phase in 2018, two coders independently checked the 885 articles with pre-determined inclusion criteria. An inter-rater reliability test revealed that the average inter-rater agreement was 87.39% (Cohen’s kappa = .61). After this, the first author (not involved in the previous inclusion check) checked the articles that previous coders classified as included by reading the articles thoroughly. Disagreements and borderline cases were discussed within the research team. The final selection check of this first phase resulted in 44 articles (see Fig. 1).

In the second data collection phase in 2020, two coders independently checked the 171 articles using the same pre-determined inclusion criteria defined in 2018. The average inter-rater agreement was 94.34% (Cohen’s kappa = .58). Disagreements and borderline cases were discussed with the research team. The final selection check of this additional phase resulted in 11 articles. After additional data collection, we obtained a final dataset consisting of 55 articles (see Fig. 1).

### Method of Analysis

Our aim was to summarize evidence of the role of online gambling and gaming communities in gambling and monetary gaming behaviors. We categorized the articles by characteristics relevant to our research: research type (quantitative or qualitative), sample characteristics, study context, topic (gambling, gaming, or both), and type of virtual community (e.g., discussion forum or in-game community). We used content analysis to summarize the main findings of the studies relevant to our research questions. Due to heterogeneity in terms of study design, participants, measures, and methods, we did not conduct a meta-analysis of the results.

## Results

### General Details About Published Studies

Studies included in the data (*n* = 55) were published between 2003 and 2020. Out of all the studies, over half (60%) were quantitative, 31% qualitative, and 9% mixed method, utilizing both quantitative and qualitative methods. Over half (60%) of the studies were gaming studies, while 35% were gambling studies, and 5% examined both gambling and gaming. In about half of the studies (48%), respondents were either from multiple countries or the study context was not explicitly mentioned. One reason for this is many of the studies utilized online surveys gathered via international online websites and forums or ethnographic data from online platforms. Regarding specific country locations, most research was conducted in Taiwan (15%), followed by Australia (7%), Finland (5%), and the United States (5%) (see Table 1). Main characteristics of the included studies are reported in Table 2.

**Table 1:** Descriptive information about the included studies (*n* = 55)

Topic	Gambling (*n *= 19)	Gaming (*n *= 33)	Both (*n *= 3)	Total (*n *= 55)
Method	% *n *= 19	% *n *= 33	% *n *= 3	% *n *= 55
Quantitative	42% (8)	70% (23)	67% (2)	60% (33)
Qualitative	42% (8)	24% (8)	33% (1)	31% (17)
Mixed method	16% (3)	6% (2)	–	9% (5)
Study context	% *n *= 19	% *n *= 33	% *n *= 3	% *n *= 55
Taiwan	5% (1)	21% (7)	–	15% (8)
Australia	21% (4)	–	–	7% (4)
Finland	16% (3)	–	–	5% (3)
Italy	5% (1)	3% (1)	–	4% (2)
UK	5% (1)	3% (1)	–	4% (2)
USA	–	9% (3)	–	5% (3)
China	–	6% (2)	–	4% (2)
South Korea	–	3% (1)	33% (1)	4% (2)
Canada, France, Malaysia	5% (1)	6% (2)	–	5% (3)
Multiple countries	16% (3)	12% (4)	–	13% (7)
Country not explicitly mentioned	26% (5)	36% (12)	67% (2)	35% (19)

**Table 2:** Main characteristics of the included studies (*n* = 55)

References	Method	Type of the study	*n*	Age	Female, %	Focus	Type of the virtual community
Badrinarayanan et al. (2014)	Quantit.	Cross-sectional	970	< 15–43+	15.0%	Gaming	MMORPG
Badrinarayanan et al. (2015)	Quantit.	Cross-sectional	970	< 15–43+	15.0%	Gaming	MMORPG
Ben-Ur et al. (2015)	Mixed	Multimethod	1695	N/A	N/A	Gaming	MMORPG; game forum
Blackburn et al. (2014)	Quantit.	Longitudinal	> 12 million	N/A	N/A	Gaming	Game community
Canossa et al. (2019)	Quantit.	Longitudinal	Multiple data	N/A	N/A	Gaming	In-game community
Caputo (2015)	Mixed	Content analytic	24	Adults	12.5%	Gambl.	PG forum
Chang et al. (2014)	Quantit.	Cross-sectional	166	< 19–40+	44.0%	Gaming	Game networks
Deans et al. (2017)	Qualit.	Interview	50	20–37	0.0%	Gambl.	SNS sites
Fang et al. (2009)	Mixed	Multimethod	33	Multiple data	Multiple data	Gaming	MMORPG
Gainsbury et al. (2015)	Qualit.	Interview	10	20–65+	40.0%	Both	SNS sites/in-game interaction
Ghazali et al. (2019)	Quantit.	Cross-sectional	362	< 18–45+	38.7%	Gaming	Game community
Gong et al. (2019)	Quantit.	Longitudinal	410	< 20–40+	29.0%	Gaming	In-game community
Goodfellow (2015)	Qualit.	Multimethod	75	N/A	N/A	Gaming	Game forums; in-game community
Griffiths and Light (2008)	Qualit.	Field study	N/A	N/A	N/A	Gaming	In-game community
Gui (2018)	Qualit.	Ethnographic	190	N/A	N/A	Gaming	Game forum; MMORPG
Hickerson and Mowen (2012)	Quantit.	Cross-sectional	141	12–38	1.0%	Gaming	In-game community
Hing et al. (2015)	Quantit.	Cross-sectional	620	Mean age 37.6	20.2%	Gambl.	PG forum/support group
Hota and Derbaix (2016)	Qualit.	Interview	20	8–12	50.0%	Gaming	MMORPG
Howe et al. (2019)	Quantit.	Cross-sectional	3361	18–88	52.0%	Gambl.	Discussion boards
Hsieh and Tseng (2018)	Quantit.	Cross-sectional	605	< 20–39	18.0%	Gaming	Online social groups
Hsu and Lu (2007)	Quantit.	Cross-sectional	356	Majority < 25	24.0%	Gaming	In-game community
Huang et al. (2012)	Quantit.	Cross-sectional	850	Majority < 24	About 33.3%	Gaming	Game forum
Huang et al. (2018)	Quantit.	Cross-sectional	2212	18–55	15.6%	Gaming	In-game community
Järvinen-Tassopoulos (2016)	Qualit.	Content analytic	48	N/A	N/A	Gambl.	PG forum
Jin et al. (2017)	Quantit.	Cross-sectional	214	< 21–36+	8.4%	Gaming	MMORPG
Kaptein et al. (2015)	Quantit.	Longitudinal	Multiple data	Multiple data	Multiple data	Both	Gambling/gaming forum
Khazaal et al. (2017)	Quantit.	Cross-sectional	372	19–61	9.0%	Gambl.	In-game interaction
Y. B. Kim et al. (2015)	Quantit.	Sentiment analytic	Multiple data	N/A	N/A	Gaming	Game forum
Y. B. Kim et al. (2017 )	Quantit.	Sentiment analytic	2,931,748	N/A	N/A	Gaming	Game forum
M. Kim and J. Kim (2018)	Quantit.	Cross-sectional	348	10–40+	14.4%	Gaming	Game community
King et al. (2020)	Quantit.	Cross-sectional	428	18–60+	6.5%	Gaming	In-game community
Lee et al. (2018)	Quantit.	Cross-sectional	320	< 20–30+	37.5%	Both	Online social networks
Lehdonvirta (2009)	Qualit.	Exploratory	N/A	N/A	N/A	Gaming	In-game community
Liao et al. (2012)	Quantit.	Cross-sectional	280	< 12–49+	19.6%	Gaming	Game community
Lin et al. (2008)	Quantit.	Cross-sectional	501	N/A	32.0%	Gaming	In-game community
Lindholm et al. (2012)	Quantit.	Longitudinal	4475	N/A	N/A	Gambl.	Poker community
McGowan (2003)	Qualit.	Discourse analytic	N/A	N/A	100.0%	Gambl.	PG forum/support group
Mudry and Strong (2013)	Qualit.	Ethnographic	1791	N/A	N/A	Gambl.	PG forum
O’Leary and Carroll (2013)	Qualit.	Ethnographic	N/A	N/A	N/A	Gambl.	Poker forum
Park et al. (2018)	Quantit.	Longitudinal	4645	N/A	N/A	Gaming	MMORPG
Parke and Griffiths (2011)	Qualit.	Ethnographic	271	N/A	N/A	Gambl.	Poker community
Pinto et al. (2015)	Qualit.	Multimethod	Multiple data	15–26	0.0%	Gaming	MMORPG
Rantala and Sulkunen (2012)	Qualit.	Content analytic	487	N/A	N/A	Gambl.	PG forum
Rapp (2018)	Qualit.	Ethnographic	Multiple data	Multiple data	Multiple data	Gaming	MMORPG
Rodda et al. (2018)	Mixed	Content analytic	1370	N/A	N/A	Gambl.	PG forum
Schüll (2016)	Qualit.	Multimethod	Multiple data	N/A	N/A	Gambl.	Poker forum; in-game interaction
Shukla and Drennan (2018)	Quantit.	Cross-sectional	358	18–35+	36.3%	Gaming	MMORPG
Sierra et al. (2016)	Quantit.	Cross-sectional	970	< 15–43+	15.0%	Gaming	MMORPG
Sirola et al. (2018)	Quantit.	Cross-sectional	1200	15–25	50.0%	Gambl.	Gambling forums (PG and other)
Sirola et al. (2019)	Quantit.	Cross-sectional	Multiple data	15–30	Multiple data	Gambl.	Gambling forums (PG and other)
Smith et al. (2012)	Quantit.	Cross-sectional	194	18–51+	2.6%	Gambl.	In-game interaction
Vella et al. (2019)	Qualitat.	Interview	36	19–30	30.56%	Gaming	In-game community
Wen et al. (2016)	Quantit.	Prototyping development	277	18–40+	36.46%	Gambl.	SNS sports betting community
Wood and Wood (2009)	Mixed	Multimethod	Multiple data	Multiple data	Multiple data	Gambl.	PG forum
Zhang et al. (2018)	Quantit.	Cross-sectional	2753	N/A	N/A	Gaming	Game community

*Qualit.* qualitative, *quantit.* quantitative, *gambl.* gambling, *PG* problem gambling, *SNS* social network site, *N/A* not applicable/not mentioned

### Online Gambling Communities

According to the reviewed studies, online gambling communities exist typically outside the game, for example, in the form of discussion forums that are created around gambling discussions. There are gambling forums for mutual gambling discussions, such as sharing gambling tips, strategies, and experiences (Howe et al. 2019; O’Leary and Carroll 2013; Parke and Griffiths 2011; Schüll 2016; Sirola et al. 2018, 2019), and also forums for sharing gambling problem experiences and discussing the downsides and related problems of gambling (Caputo 2015; Hing et al. 2015; Järvinen-Tassopoulos 2016; McGowan 2003; Mudry and Strong 2013; Rantala and Sulkunen 2012; Rodda et al. 2018; Sirola et al. 2018, 2019; Wood and Wood 2009). In addition, there are also some in-game interactional tools, such as chat opportunities, for gamblers, particularly in online poker (Khazaal et al. 2017; Schüll 2016; Smith et al. 2012) and in online social casino games (Gainsbury et al. 2015).

Participation in online communities with positive gambling attitudes is a risk factor for excessive gambling (Howe et al. 2019; Sirola et al. 2018, 2019). A study by Sirola et al. (2019) found that sense of loneliness moderated the association between excessive gambling and daily online gambling community participation in Finland, indicating that lonely problem gamblers are most likely to actively participate in such communities. Online poker communities are mostly used for sharing poker experiences and seeking social reinforcement for gambling successes; these kinds of communities may also increase poker playing and help develop cognitive biases concerning gambling (Parke and Griffiths 2011). However, there was also some evidence that actively participating in mutual discussion in a gambling community and actively consuming money in online gambling are mutually exclusive activities (Kaptein et al. 2015; Lindholm et al. 2012). Using longitudinal data of online poker players, it was noticed that when consumers increased their community activity, they also reduced their poker-related consumption (Lindholm et al. 2012). In addition, when relatively inactive community members increased their community activity, it was related to increased money consumption, while already active members’ increase in community engagement was related to decreased spending (Kaptein et al. 2015).

Online poker players share their poker data and experiences of former games with other poker players in online forums, chat threads, and message boards to get feedback and help to identify flaws in performance; this may also protect from overvaluing one’s poker skills (Schüll 2016). Feedback from the community members is considered helpful in developing one’s poker skills, and it may even reduce the risk of problematic gambling, as long as the information provided is accurate (Parke and Griffiths 2011). In addition, socializing with other players during online gambling by utilizing in-game interaction tools is associated with less problematic forms of gambling (Khazaal et al. 2017; Smith et al. 2012). A study by Khazaal et al. (2017) found that gambling problems were more severe among lonely online gamblers who did not utilize social interaction tools in a game or preferred to gamble against the computer. Thus, it seems that in online poker, utilizing poker communities both in- and outside the game may protect the player from developing excessive poker gambling habits.

Although communities may offer safeguards for poker players, research shows that gambling-related social networks and exposure to the gambling activities of peers may normalize gambling and make it attractive. Gambling-related activities of Facebook friends, such as “liking” social casino games and inviting friends to play, influence users’ intentions to try these gambling or gambling-like activities (Gainsbury et al. 2015). In mobile social-network games, the perceived number of users and friends increases the jackpot and purchase intentions of probability-based items (Lee et al. 2018). In online sports betting communities, users prefer sharing personal betting results and wagering opinions and predictions with others (Wen et al. 2016). Users can also extend their gambling-related networks to share wagering tips and celebrate wins with others; these kinds of gambling-positive discussions may contribute to the normalization of gambling (Deans et al. 2017).

Communities focusing on gambling problems have essential roles for those coping with problematic gambling; they may even help with overcoming problems. Discussions on gambling problem forums are grounded in sharing gambling problem experiences and related problems (Caputo 2015; Järvinen-Tassopoulos 2016; Rantala and Sulkunen 2012), and also strategies for getting rid of gambling problems (Rodda et al. 2018). From a user’s perspective, these kinds of communities are important sources of mutual support, by helping him or her to better cope with gambling problems and to feel less alone with his or her problems (Wood and Wood 2009). However, a survey study from Finland on young respondents aged 15–25 found that the main motivation for respondents to engage in online gambling communities was to share gambling tips and general gambling information, while only a few mentioned discussing gambling problems and recovery (Sirola et al. 2018). Also, a study by Hing et al. (2015) found that online problem gamblers were more reluctant to utilize online support groups or discussion boards compared to land-based problem gamblers.

Gambling communities are grounded on mutual norms, where it is important to conform in order to be accepted as a legitimate member of the community (Mudry and Strong 2013; O’Leary and Carroll 2013). Communities are also important for a gambler’s identity; poker forums are spaces to construct poker player identities (O’Leary and Carroll 2013), but online communities focused on problem gambling can also be utilized in negotiating and (re)constructing problem gambler identities (Järvinen-Tassopoulos 2016; Mudry and Strong 2013).

There was also some evidence of gender-specific differences in the use of online gambling communities. In a study by Khazaal et al. (2017), women were less prone to utilize in-game interaction tools; this could be at least partly explained by the male-dominance typically associated with gambling. Since gambling problems have traditionally been more common among males than females, online forums offer a space for female problem gamblers to anonymously share their gambling problem experiences (Järvinen-Tassopoulos 2016; McGowan 2003; Wood and Wood 2009), which can be challenging or intimidating in male-dominated face-to-face groups (McGowan 2003). Also, in a study by Wood and Wood (2009), significantly more women than men found gambling problem forums helpful in coping with their gambling problem.

### Online Gaming Communities

According to reviewed studies, online gaming communities inherently exist inside the game. This is especially true with MMORPGs (Badrinarayanan et al. 2014, 2015; Ben-Ur et al. 2015; Fang et al. 2009; Gui 2018; Hota and Derbaix 2016; Jin et al. 2017; Park et al. 2018; Pinto et al. 2015). MMORPG playing typically takes place in guilds, that is, long-lasting social groups where players collaborate in order to better game success. In guilds, players share their skills, knowledge, and virtual resources, such as money, with each other (Gui 2018; Pinto et al. 2015). The player roles in guilds are important in terms of teamwork contributions. An example of this type of contribution would be taking care of a guild bank that is used for sharing common resources, like items and money (Rapp 2018). Social interaction with other players is one of the motivating factors in playing (Fang et al. 2009), and it may also have positive outcomes for a player’s social capital. Indeed, a study by Hickerson and Mowen (2012) found that gamers who utilized social bonding in video games reported positive social outcomes, such as friend-based social support.

Perceived group cohesion is an important determinant in a user’s preference for participating in an online game community, and a community’s social norms can affect a customer’s loyalty towards the community (Hsu and Lu 2007). Ben-Ur et al. (2015) suggested that a strong virtual game community intensifies hedonic consumption experience and satisfaction among its members. Lin et al. (2008) found that women are more likely than men to commit to a game if it utilizes interactional tools to create and maintain social relationships with other gamers; this was also associated with consumer satisfaction and loyalty. According to  M. Kim and J. Kim (2018), financial incentives (e.g. special price offerings or rewards) in an online game community, alongside with social and structural bonds, play an important role in users’ online community engagement.

Various studies indicated that a game community, either in-game or out-game, has an important role in terms of purchase intentions and consumption behavior within a game. Huang et al. (2018) found that gamers’ interdependence (i.e. depending on other players’ opinions) and network convergence (i.e. shared friends with other players) were positively related to continuance intention. A study by Zhang et al. (2018) found that players’ sense of community in game communities is positively associated with purchase behavior. In a study of Pokémon Go users by Ghazali et al. (2019), discussing the game and sharing experiences in a virtual game community enhanced gaming experience, and online community involvement mediated the relationship between network externality and continuance intention. In terms of MMORPG communities, studies utilizing structural equation modeling illustrated that identifying with a specific MMORPG community drives purchase intention and consumption behavior (Badrinarayanan et al. 2014, 2015). Sierra et al. (2016) found that becoming attached to a MMORPG community intensifies a player’s tribal psyche associated with the MMORPG, which in turn enhances self-esteem. Further, self-esteem positively influences virtual purchase intentions within the MMORPG. A study by Canossa et al. (2019) indicated that game networks have a social contagion effect in a way that certain active players serve as influencers in a gaming network. These influencers then impact other players’ gaming habits, such as time and money invested in a game, and social play with others (Canossa et al. 2019).

Studies also examined the role of social influence in gaming communities in terms of virtual purchases. According to Hsieh and Tseng (2018), online informational influence (i.e., relying on online peers’ knowledge of online games and virtual items) directly affects intentions to buy virtual items, and this relationship was also mediated by happiness. In a study by Shukla and Drennan (2018), it was found that normative interpersonal influence (i.e., conformity in order to be approved by peers) and community identity within the MMORPG community influence virtual purchase intentions. In a study by Chang et al. (2014), peer-influence was positively associated with subjective norm, and subjective norm was further positively related to continuance intention to play online games. Park et al. (2018) found that social interaction between users in a MMORPG community positively affects both hedonic and functional product purchases, but social influence has a stronger impact on consumption of hedonic rather than functional products. Hota and Derbaix (2016) found that even 8–12-year-old children utilize teamwork aspects in their gaming and are susceptible to peer influence in virtual consumption. Observed gaming behavior and social norms of other players may influence excessive gaming behavior through social learning mechanisms (Gong et al. 2019). A study by King et al. (2020) found that in a highly popular online game Fortnite, spending on microtransactions was influenced by in-game friends’ purchase behavior. In addition, those who belonged to a larger online social network of Fortnite players were likely to spend money on microtransactions.

The motives for buying virtual items in online games are functional, hedonistic, and social; virtual items have social value, for example, in terms of social distinction and status (Lehdonvirta 2009). Interviews with 8–12-year-old children revealed that boys prefer buying virtual items for better game performance, while girls buy items for social status (Hota and Derbaix 2016). According to Gong et al. (2019), young gamers who play excessively spend lots of money on in-game purchases, which can lead to conflicts with family members.

Players help each other in virtual game communities by giving tips to better game performance (Ben-Ur et al. 2015; Hota and Derbaix 2016), sharing knowledge of the virtual products (Hota and Derbaix 2016), and recommending suitable and discounted games for others (Ben-Ur et al. 2015; Vella et al. 2019). Symbolic customer value, such as group membership in a game community, positively affects purchase intentions and likelihood to recommend products or services in online word-of-mouth communications (Liao et al. 2012). In a study by Huang et al. (2012), a sense of virtual community moderated the influence of other users’ comments on attitudes and purchase intentions.

Membership of a guild becomes an important and extended part of the identity, which becomes manifested in game-related consumption (Pinto et al. 2015). Both technological (i.e., interactivity, social presence) and user factors (i.e., social ties, social identity) have strong positive relationships with the users’ purchase intentions; further, social ties and social identities affect user engagement and community satisfaction (Jin et al. 2017).

MMORPGs and their guild-systems are characterized by shared roles (Rapp 2018) and mutual norms and policies concerning acceptable gaming behaviors. Malicious and grief (i.e., impolite and unethical) players are perceived as threatening to the community and its playing policies (Hsu and Lu 2007). Cheating and scamming in order to gain monetary benefits and virtual items are seen as norm-breaking and are socially sanctioned behaviors within game communities (Blackburn et al. 2014; Goodfellow 2015). However, in some game communities, such as in Habbo Hotel, scamming and cheating are regarded as normal and harmless activities despite their antisocial nature (Griffiths and Light 2008).

In addition to in-game communities, there are also game-related discussion forums where gamers can interact (Ben-Ur et al. 2015; Goodfellow 2015; Gui 2018; Huang et al. 2012; Y. B. Kim et al. 2015, 2017). Game forums are important platforms for gamers to share experiences of games, and this kind of word-of-mouth communication may also affect game purchase intentions (Huang et al. 2012). In game review forums, gamers give recommendations of games for other players (Ben-Ur et al. 2015). In game-specific discussion forums, gamers can discuss all the things related to a specific game and, for example, criticize other players’ playing strategies and habits (Goodfellow 2015). Gamers also share their opinions of in-game virtual currencies in game-specific discussion forums, and even currency value fluctuations can be predicted based on these user opinions (Y. B. Kim et al. 2015, 2017).

### Similarities and Differences Between Online Gambling and Gaming Communities

Online gambling and gaming communities have both differences and similarities regarding characteristics, reasons of use, and outcomes of use (see Table 3). In gambling studies, online communities are typically discussion forums and other virtual spaces that exist outside a game (Caputo 2015; Hing et al. 2015; Howe et al. 2019; Järvinen-Tassopoulos 2016; McGowan 2003; Mudry and Strong 2013; O’Leary and Carroll 2013; Parke and Griffiths 2011; Rantala and Sulkunen 2012; Rodda et al. 2018; Schüll 2016; Sirola et al. 2018, 2019; Wood and Wood 2009), but also some in-game interaction tools exist particularly in online poker (Khazaal et al. 2017; Schüll 2016; Smith et al. 2012) and in social casino games (Gainsbury et al. 2015). Gaming communities, on the other hand, exist inherently embedded inside the game, as is the case particularly in MMORPGs and their guild-based systems (Badrinarayanan et al. 2014, 2015; Ben-Ur et al. 2015; Fang et al. 2009; Gui 2018; Hota and Derbaix 2016; Jin et al. 2017; Park et al. 2018; Pinto et al. 2015), but also external communities such as discussion forums exist for gamers (Ben-Ur et al. 2015; Y. B. Kim et al. 2015, 2017). Strikingly, at least within this data, no gaming problem forums or communities were identified, as was the case with gambling.

**Table 3:** Summarizing key differences and similarities between gambling and gaming communities in relation to gambling and monetary gaming

	Gambling	Gaming
External communities	Gambling-related discussion forums (e.g., for discussions on gambling in general, gambling tips and problem gambling)	Game-specific discussion forums, game review forums
In-game communities	In-game interaction tools in online poker and social casino games	Community aspect inherently embedded (e.g., in MMORPGs)
	In-game community aspect not central	
Community role in gambling/monetary gaming	Normalize gambling and involve risks for excessive gambling	In-game interaction is an essential part of gaming experience
	Feedback from the community helps to develop poker skills	Identifying with a gaming community drives game-related purchase intentions
	Reinforce gambler identities	Gamers help each other to better game performance and provide information of virtual items
	Utilizing in-game interaction is associated with non-problematic forms of gambling	Reinforce gamer identities
Community role in problematic gambling/monetary gaming	Critical feedback from the community may protect from maladaptive gambling habits	Not examined in the included studies
	Peer-support in gambling problem forums helps to cope with one’s gambling problem and aiming for recovery	
	Important in (re)constructing problem gambler identities	

Mutual for both gambling and gaming communities is the importance of their community-specific norm system; being accepted as a legitimate member of the community requires following and conforming to the community’s norms (Blackburn et al. 2014; Goodfellow 2015; Griffiths and Light 2008; Gui 2018; Mudry and Strong 2013; O’Leary and Carroll 2013). Both gambling and gaming communities are also important in gambling- and gaming-related identity constructions (Järvinen-Tassopoulos 2016; Mudry and Strong 2013; O’Leary and Carroll 2013; Pinto et al. 2015).

According to the studies reviewed, utilizing in-game interaction and socializing with other players during the game have different functions and outcomes in online gambling and gaming. In gaming studies, there is strong evidence that identifying with in-game communities has a great potential to influence gaming behavior and in-game purchase intentions (Badrinarayanan et al. 2014, 2015; Canossa et al. 2019; Ghazali et al. 2019; Gong et al. 2019; Hota and Derbaix 2016; Hsieh and Tseng 2018; Huang et al. 2018; King et al. 2020; Park et al. 2018; Shukla and Drennan 2018; Sierra et al. 2016; Zhang et al. 2018). In gambling studies, on the contrary, there is evidence that socializing with other players during a game, particularly in online poker, might be a protective factor, as this kind of social playing was associated with less severe and non-problematic forms of gambling (Khazaal et al. 2017; Smith et al. 2012). In general, it seems that social motives are more inherently embedded in video gaming compared to online gambling. For example, when interviewing players of social casino games (i.e., gambling-like online games), few of the interviewees mentioned playing for social motives, despite the interactional opportunities of the game (Gainsbury et al. 2015); while in video gaming, social interaction with other players is considered an important motive for playing (Fang et al. 2009; Hickerson and Mowen 2012).

Studies also indicate differences concerning a community’s potential protective role and feedback in terms of excessive gambling or gaming habits. In gambling studies, there was evidence that feedback from an online gambling community could influence gambling behavior to a more moderate direction and protect from overvaluing one’s poker skills (Parke and Griffiths 2011; Schüll 2016). There was also some evidence that actively contributing in an online gambling community could decrease gambling-related consumption (Kaptein et al. 2015; Lindholm et al. 2012). On the contrary, there were no studies or results indicating a gaming community’s protective role or critical feedback concerning excessive gaming or in-game purchase behaviors. Instead, studies consistently showed the motivating effect of a gaming community in terms of gaming continuation and purchase intentions.

There was also some evidence concerning gender differences in the use of virtual gambling and gaming communities. In online poker, females did not prefer using in-game interaction tools, while men did (Khazaal et al. 2017). Instead, women with a gambling problem found discussion forums important in coping with their gambling-related problems (Järvinen-Tassopoulos 2016; Wood and Wood 2009). In gaming studies, Lin et al. (2008) found that women were more likely than men to commit to a game if it provided tools to create and maintain social relationships. However, since the proportion of female participants in the reviewed studies was significantly smaller compared to males, evidence of potential gender differences remains weak.

## Discussion

The aim of this review was to summarize research on online gambling and gaming communities and their role in gambling and monetary gaming behaviors. In total, 55 articles filled the criteria; 60% of them were quantitative, and the rest were either qualitative or mixed method. Out of the articles, 33 were on gaming, 19 on gambling, and only three studies investigated both gambling and gaming. Despite a relatively limited number of studies on this area, the results show that identification with virtual communities has an influential role in gambling and monetary gaming behaviors, but there were also some notable differences in community types and possible outcomes of the community use between gambling and gaming communities.

In line with research on online identity formation (Kaakinen et al. 2020; McNamara and Parsons 2016), results show that virtual communities are important spaces for gamblers and gamers to construct and extend their identities concerning gambling and gaming with like-minded others. In MMORPGs, virtual game communities are grounded on collaboration, teamwork, and mutual goals, and the communities can become an extended part of the identity. In gambling, poker communities are important spaces for poker players to enhance their poker player identities via social reinforcement and community feedback. For problem gamblers, there are virtual communities to share their experiences with other problem gamblers and receive socio-emotional peer support for dealing with problems. Various studies of this review also pointed out the role of social influence in both gambling and gaming communities, for example, in terms of purchase intentions and trying out new gambling activities. Normalizing and promoting gambling and gambling-like activities in social media can make gambling attractive and encourage excessive gambling habits via social influence and perceived norms (e.g., Cialdini and Goldstein 2004).

One notable difference of gambling and gaming communities concerned the communities’ roles in game-related money use and purchase intentions. Whereas studies suggested that feedback from gambling communities can also protect from developing excessive gambling habits, gaming communities seem to solely motivate gaming behaviors and purchase intentions. A possible explanation for the differences is the fundamentally different nature of gambling compared to gaming. Succeeding in gambling, in terms of winning money, is highly individual by nature. Thus, members of a gambling community may be more prone to notice and criticize potentially problematic gambling behavior, as no one else of the community shares the benefits of the gambling success or money invested in gambling other than the gambler. In video gaming, in contrast, success in game and money invested for it could also benefit the community teammates, particularly in MMORPGs where gaming is typically formed around guilds. In other words, if committed to teamwork play, purchasing virtual items are for the community’s good and not solely for the individual’s. Thus, even excessive gaming and money use within the game can be important in terms of a team’s performance and success in the game. This makes it unlikely that members of the community would try to restrain their team players’ gaming activity because it would mean poorer game performance for the team.

Differences also existed concerning the role of in-game interaction. Although both digital games and online gambling games include in-game interaction tools, the role of in-game socialization in gambling and gaming proved to be inherently different. Indeed, it can be suggested based on the results that in online gambling lonely gamblers who do not socialize with other gamblers are more prone to use more money and to develop more severe gambling problems; in other words, social playing was associated with non-problematic forms of gambling. In video gaming, on the other hand, playing in isolation may result in less purchase intention within a game, since identifying with a game community was consistently and positively associated with in-game purchase intentions. Thus, the roles of social interaction and social influence should be taken into consideration when screening for potentially problematic forms of gaming behavior.

It is also noteworthy that while in gambling studies, there were forums for those seeking help for and sharing experiences of gambling problems, there were no studies on communities of problematic gaming in our data. A plausible explanation for the lack or scarcity of these communities is that there is a general lack of consensus on the phenomenon and definition of problematic gaming and whether it can be qualified as an addiction (Griffiths et al. 2015). Recently, “gaming disorder” has been included in the latest International Classification of Diseases (ICD-11), and in the fifth edition of the Diagnostic and Statistical Manual of Mental Disorders (DSM-V), it is recognized as a condition that requires more research before including it into mental disorders. The proposal of gaming disorder as a diagnosis has aroused a great deal of criticism among scholars due to, for example, low quality of the research base and problems in operationalization (Aarseth et al. 2017). However, it may be that if gaming disorder becomes established in general discourses and addiction treatments, gaming problem forums and online self-help groups will become more common.

From a theoretical perspective of virtual communities, the results of this systematic review show that virtual communities in gambling and gaming are grounded on mutual goals, shared interests, and norms. These aspects have been previously noted in studies on online communities (Boellstorff 2015; Preece 2000; Oksanen et al. 2014), and these communities play an important yet different role for gamblers and gamers. Despite some notable differences between gambling and gaming communities, it is clear that both types of communities provide their users virtual spaces to fulfill a fundamental need to belong and form social ties (Baumeister and Leary 1995; Deci and Ryan’s 2000). Virtual social ties may be valuable for those who have deficient offline relationships, and socialization with online friends is also a significant part of the fun, particularly in video gaming, and may have positive outcomes for a player’s social capital. However, this systematic literature review emphasizes the risks involved. It particularly recognizes the impact communities have, through social mechanisms, on monetary behavior and other potentially harmful consequences. Based on the results, we highlight that more emphasis should be placed in examining online communities’ roles in problematic gambling and gaming habits, particularly in terms of excessive money consumption.

### Limitations

This study is not without its limitations. First, it is possible that some relevant articles have been excluded in the search phase due to the search words used. Second, in terms of gaming phenomena, we limited our focus on studies examining gaming with explicitly mentioned monetary behavior. Although microtransactions and gambling-like mechanisms are common business models in the majority of digital games, we did not include studies where monetary behavior was not explicitly mentioned. Online gaming communities and social interactions within them may play various important roles for gamers in general, but this review only focused on a community’s role on monetary gaming behavior, such as virtual purchase intentions. Finally, in this review we studied virtual gambling and gaming communities as factors in gambling and monetary gaming behaviors. Thus, this review does not cover those forms of gambling- or gaming-related virtual interactions and communities whose relationship to actual gambling or gaming behavior remains unstudied.

### Conclusion

Although online gambling and gaming are isolated activities in the sense that the player is often physically alone, related virtual communities are an essential part of both activities. Online gambling and gaming communities normalize gambling and gaming behaviors and influence purchase intentions; but at least in gambling, communities may also support moderate forms of gambling, provide socio-emotional support for recovery of addiction and help to cope with a gambling problem. Even though the line between gambling and gaming has become blurred due to increased use of gambling-like mechanisms in digital games, the results of this review indicate that social interactions in these two activities have different functions, and also motives for and outcomes of the interaction differ in terms of monetary behavior.

The role of virtual communities should be acknowledged in prevention and treatment of gambling and gaming problems. In particular, it would be crucial to understand social mechanisms, such as social influence and social learning, taking place in virtual gambling and gaming environments. Raising awareness of social underpinnings and influential mechanisms behind gambling and monetary gaming would be important for players, parents and health care professionals when aiming to reduce excessive behavior and money consumption. Limiting players’ in-game social interaction would be required to reduce excessive money spending, particularly in group- and guild-based gaming, where purchase intention often follows strong belonging or attachment to the community. In gambling, utilizing recovery-oriented virtual communities for problem gamblers would be useful given that such communities are proven to be useful in implementing beneficial aspects of peer-influence, support and anonymity. Finally, improving gamblers’ and gamers’ offline relationships and healthy activities would be crucial in risk-prevention. Meaningful offline relationships and social activities would decrease the need for spending lots of time gambling and gaming online, but also diminish the need for belonging to virtual communities and searching for social contacts online.
